# Supplementary material for: COVID-19 Vaccine Hesitancy—A Scoping Review of Literature in High-Income Countries
Source: Vaccines (Basel). 2021 Aug 13;9(8):900. doi: 10.3390/vaccines9080900 (PMC8402587; doi:10.3390/vaccines9080900)
Supplement: Supplementary file 1 [file vaccines-09-00900-s001.zip › vaccines-1277111-supplementary.pdf]

Supplementary Table S1. Detailed search strategy

S/N	Key Themes	Search details
**Medline**		
**S/N**	**Key Themes**	**Search details**
1	COVID-19 vaccine	(("vaccination refusal"[MeSH Terms] OR "hesitancy"[Title/Abstract] OR "confidence"[Title/Abstract] OR "accept*"[Title/Abstract] OR "doubt"[Title/Abstract] OR "uncertainty"[Title/Abstract] OR "indecision"[Title/Abstract] OR "sceptic*"[Title/Abstract] OR "reluctance"[Title/Abstract] OR "delay"[Title/Abstract] OR "reservation*"[Title/Abstract] OR "ambivalence"[Title/Abstract] OR "unwilling*"[Title/Abstract] OR "hesit*"[Title/Abstract] OR "uptake"[Title/Abstract] OR "decision"[Title/Abstract] OR "adherence"[Title/Abstract] OR "compliance"[Title/Abstract] OR "willingness"[Title/Abstract] OR "concerns"[Title/Abstract])
2	Hesitancy	("covid 19 vaccines"[MeSH Terms] OR "covid 19 vaccin*"[Title/Abstract] OR "covid vaccin*"[Title/Abstract] OR "sars cov 2 vaccin*"[Title/Abstract] OR "severe acute respiratory syndrome vaccin*"[Title/Abstract])) OR "coronavirus vaccin*"[Title/Abstract]
Search strategy: #1 AND #2		
**CINAHL**		
**S/N**	**Key Themes**	**Search details**
1	COVID-19 vaccine	MH COVID-19 vaccines OR AB covid-19 vaccin* OR AB covid vaccin* OR AB sars-cov-2 vaccin* OR AB severe acute respiratory syndrome vaccin* OR AB coronavirus vaccin*
2	Hesitancy	MH vaccination refusal OR AB hesitancy OR AB confidence OR AB accept* OR AB doubt OR AB uncertainty OR AB indecision OR AB sceptic* OR AB reluctance OR AB delay OR AB reservation* OR AB (ambivalence OR unwilling* OR hesit* OR uptake OR decision OR adherence OR compliance OR willingness OR concerns)
Search strategy: #1 AND #2		
**EMBASE**		
**S/N**	**Key Themes**	**Search details**
1	COVID-19 vaccine	'sars-cov-2 vaccine'/exp OR 'covid-19 vaccin*':ab,ti OR 'covid vaccin*':ab,ti OR 'sars-cov-2 vaccin*':ab,ti OR 'severe acute respiratory syndrome vaccin*':ab,ti OR 'coronavirus vaccin*':ab,ti
2	Hesitancy	'vaccination refusal'/exp OR hesitancy:ab,ti OR confidence:ab,ti OR accept*:ab,ti OR doubt:ab,ti OR uncertainty:ab,ti OR indecision:ab,ti OR sceptic*:ab,ti OR reluctance:ab,ti OR delay:ab,ti OR reservation*:ab,ti OR ambivalence:ab,ti OR unwilling*:ab,ti OR hesit*:ab,ti OR uptake:ab,ti OR decision:ab,ti OR adherence:ab,ti OR compliance:ab,ti OR willingness:ab,ti OR concerns:ab,ti
Search strategy: #1 AND #2		
**Scopus**		
**S/N**	**Key Themes**	**Search details**
1	COVID-19 vaccine	((TITLE-ABS-KEY (covid-19 AND vaccin*) OR TITLE-ABS-KEY (covid AND vaccin*) OR TITLE-ABS-KEY (sars-cov-2 AND vaccin*) OR TITLE-ABS-KEY (severe AND acute AND respiratory AND syndrome AND vaccin*) OR TITLE-ABS-KEY (coronavirus AND vaccin*)))
2	Hesitancy	((TITLE-ABS-KEY (vaccination AND refusal) OR TITLE-ABS-KEY (hesitancy) OR TITLE-ABS-KEY (confidence) OR TITLE-ABS-KEY (accept*) OR TITLE-ABS-KEY (doubt) OR TITLE-ABS-KEY (uncertainty) OR TITLE-ABS-KEY (indecision) OR TITLE-ABS-KEY (sceptic*) OR TITLE-ABS-KEY (reluctance) OR TITLE-ABS-KEY (delay) OR TITLE-ABS-KEY (reservation) OR TITLE-ABS-KEY (ambivalence) OR TITLE-ABS-KEY (unwilling*) OR TITLE-ABS-KEY (

	hesit*) OR TITLE-ABS-KEY (uptake) OR TITLE-ABS-KEY (decision) OR TITLE-ABS-KEY (adherence) OR TITLE-ABS-KEY (compliance) OR TITLE-ABS-KEY (willingness) OR TITLE-ABS-KEY (concerns)))
Search strategy: #1 AND #2	

Table S2. Characteristics of included studies.

**Author**	**Country**	**Study design and methodology**	**Sample size**	**Age**	**Participants type**	**Gender (Male, %)**	**Education**	**Vaccine hesitancy rate (%)**	**Hesitancy defined as per SAGE recommendations (Y/N/NS)**
COCONEL GRP: Peretti-Watel et al 2020 (28)	France	XS Online survey	3892	NS	General public	NS	NS	26	N
Al-Mohaithef et al 2020 (62)	Saudi Arabia	XS Online survey	992	Age 18-25: 26.6%<br>Age 26-35: 44.0%<br>Age 36-45: 24.1%<br>Above 45: 5.1%	General public	34.2	Postgraduate: 20.1%<br>Graduate: 50.1%<br>Diploma: 15.4%<br>High School: 14.4%	35	Y
Alabdulla et al 2021 (20)	Qatar	XS Online survey	7821	Age 18 - 25: 3.3%<br>Age 26 - 35: 31.9%<br>Age 36 - 45: 34.1%<br>Age 46 - 55: 15.0%<br>Age 56 - 65: 11.6%<br>Above 65: 4.2%	General public	59.4	Graduate: 76.8%<br>Others: 12.8%<br>High school: 10.4%	40<br>42.2^	Y
AlHajri et al 2020 (19)	Kuwait	XS Online survey	1038	NS	General public	NS	NS	55.8^	NS
Alley et al 2021 (78)	Australia	MM Online survey	2343	Survey 1:<br>18–34: 13.7%<br>35–44: 15.8%<br>45–54: 19.7%<br>55–64: 28.7%<br>65 and over: 22.1%<br>Survey 2:	General public	Survey 1: 30.4<br>Survey 2: 30	Survey 1:<br>Year 12 or below: 11.1%<br>Technical studies, certificate, diploma: 25.4%<br>Bachelor and above:	14	N

				Over 55 years old: 51%			63.5% Survey 2: Bachelor and above: 63%		
Amin et al 2021 (74)	USA	XS Online survey	240	Median age: 41 - 50	Healthcare workers	43	NS	8	N
Attwell et al 2021 (29)	Australia	XS Online survey	1316	Mean 57.95 (SD 13.2)	General public	40	Mean yrs /SD: 13.70/ 3.43	35	Y
Barello et al 2020 (101)	Italy	XS Online survey	934	Mean age 23.6 (SD 4.9)	University students	20.4		13.9	Y
Barrière et al 2021 (30)	France	XS Paper survey	999	Median age: 67 (IQR 18 -97)	Cancer patients	43.9	NS	46.3	Y
Beesley, et al 2021 (21)	Multiple countries	Longitudinal prospective study Online survey	1645	Not specified	Patients with autoimmune conditions	Not specified	NS	13 34^	Y
Bell, S. 2020 (23)	England	MM Online survey and telephone interview re: qualitative component#	1271	Mean 33.0 (SD 4.57)	Parents	5 (97% of female respondents have a partner)	NS	9.9 10.9^	Y
Benham, J. L. 2021 (26)	Canada	Qualitative#	50	Age 18–29 years: 34% Age 30–39 years: 12% Age 40–49 years: 8% Age 50–59 years: 20% Age 60 and over: 26%	General public	40 (n = 20)	NS	NS	NS
Bogart et al 2021 (79)	USA	XS Telephone interview	101	Mean 50.3 (SD 11.5)	General public	87	Education level less than high school: 13%	32	N
Bokemper Et al 2020 (94)	USA	RCT via online methods	5014	NS	General public	NS	NS	Measured acceptance as a continuu	N

								m from 0-1	
Borriello et al 2020 (110)	Australia	XS Online survey	2136	Median age: 59 (IQR: 31-76)	General public	49.3	Year 10 or below: 10.58%<br>Year 11 and 12: 15.03%<br>Certificate I or II: 1.83%<br>Certificate III or IV: 9.04%<br>Advanced Diploma or Diploma: 13.62%<br>Bachelor degree and above: 42.28% (disproportionately more degree holders than in Aust representative pp of 22%)<br>Other: 7.63%	Discrete choice experiment to measure acceptability to 3 choices of vaccines with different characteristics	NS
Caban-Martinez et al 2021 (63)	USA	XS Online survey	3169	21 – 29: 7.2%<br>30 – 39: 31.3%<br>40 – 49: 32.9%<br>50 – 59: 24.2%<br>60 years: 4.4%	Firefighters	92.5	High School/GED: 6.5%<br>Some College: 43.0%<br>College Graduate: 50.5%	51.8	N
Callaghan et al 2021 (31)	USA	XS Online survey	5009	Weighted Mean age: 45.9	General public	48.5 (weighted)	College degree: 34.3% (weighted)	31.1	N
Campochiaro et al 2021 (80)	Italy	Case control self-administered paper questionnaire survey	472	Rheumato (case)<br>Age n (%)<br>18–24 5 (2)<br>25–54 83 (41)<br>55–64 45 (22)<br>>65 69 (34)<br>Age (years), n (%)<br>Onco (control)<br>Age n (%)<br>18–24 0 (0)	Patients with autoimmune conditions	Rheumato 35.6 (n=96)<br>Onco 14.7 (n=38)	NS	Rheumato: 17<br>Onco: 9	Y

				25–54 12 (18)<br>55–64 21 (31)<br>>65 35 (51)					
Caserotti et al 2021 (103)	Italy	Longitudinal prospective Online survey	3691	38.1/14	General public	30.1	NS	59.9	Y
Chu et al 2021 (98)	USA	XS Online survey	934	46 / 16.2	General public	48.7	Median: 4-year college degree	Measured acceptance on a continuum scale mean of responses to 4 qns with max score of 5: (3.98 / 1.25)	N
Detoc et al 2020 (32)	France	XS Online survey	3259	Under 30 years: 20.6%<br>30–49: 46.1%<br>50–64: 24.6%<br>65–80: 8.3%<br>Over 80: 0.4%	General public	32.6	NS	22.4	Y
Dodd et al 2020 (64)	Australia	XS Online survey	4362	18-25 years: 22.1%<br>26-40 years: 27.9%<br>41-55 years: 22.0%<br>56-90 years: 28.0%	General public	NS	High school or less: 21.4%<br>Certificate I-IV: 14.1%<br>University: 64.4%	14.3	Y
Dror et al 2020 (15)	Israel	XS Online survey	2770	Not specified	General public	NS	NS	General public: 22<br>Nurses: 39<br>Drs.: 25	N

								Mean: 28 On vaccinating their children^: 30, 45, 40 respectively	
Ehde et al 2021 (81)	USA	XS Online survey	486	55.7/12.6	General public	17.3	< 12th grade: 0.4%<br>High school graduate or GED: 4.7%<br>Vocational or Technical School: 5.8%<br>Some college: 19.5%<br>College graduate: 38.1%<br>Graduate or professional school: 31.5%	15.4	N
Faasse et al 2020 (65)	Australia	XS Online survey	2232	18–29: 22.5%<br>30–49: 39.4%<br>50–59: 22.4%<br>60+: 13.9%<br>Not stated: 1.7%	General public	23.1	High school only(completed (Year 12) or not completed (Year 11 or below): 24.6%<br>Trade certificate, diploma, or advance diploma: 24.3%<br>Bachelor's degree: 25.9%<br>Graduate diploma, graduate certificate, or postgraduate degree: 25.0%<br>Not stated: 0.3%	18.8	Y

Feleszko et al 2021 (33)	Poland	XS Online survey	1066	18–24 11.7%<br>25–34 22.8%<br>35–44 23.8<br>46–65 41.7%	General public	49.8	NS	62	Y
Fisher et al 2020 (66)	USA	MM telephone, mail, in-person face to face interviews	991	48.0/18.1	General public	48.5	No high school diploma: 9.7%<br>High school graduate or equivalent: 28.2%<br>Some college: 27.6%<br>College graduate or above: 34.5%	42.4	Y
Freeman et al 2020 (105)	England	XS Online survey	2501	46.6 / 17.3	General public	48.5	No qualifications: 4.8%<br>GCSEs grades A*–C (or equivalent): 24.8%<br>AS levels (or equivalent): 3.9%<br>A levels (or equivalent): 24.0%<br>Certificate of higher education (e.g. BA, BSc, or equivalent): 30.5%<br>Post-graduate qualifications (e.g. MA, MSc, PhD, DPhil): 12.1%	12.1	Y
Freeman et al 2020 (34)	U.K.	XS Online survey	5114	46.9 (17.1)	General public	50.3	No qualification: 6.5%<br>GCSEs grades A*−C (or equivalent): 26.6%<br>AS levels (or equivalent): 5.0%<br>A levels (or equivalent): 26.5%<br>Certificate of higher education e.g.BA, BSc, or equivalent): 24.6%<br>Post-graduate qualifications (e.g.	23.9	N

Gadoth et al 2021 (67)	USA	XS Online survey, with open ended questions	1069	18–29: 16.1%<br>30–39: 40.0%<br>40–49: 23.0%<br>50–59: 13.9%<br>≥60: 7.0%	Healthcare workers	28.3	MA, MSc, PhD, DPhil): 11.0%<br>Job role:<br>Prescribing clinicians: 37.2%<br>Nurses: 38.3%<br>Other personnel with direct patient contact: 9.8%<br>Personnel without patient contact: 14.6%	66.8	Y
Gagneux-Brunon et al 2020 (35)	France	XS On site paper questionnaire	2047	Under 30: 22.7%<br>30 - 49: 47.3%<br>50 - 64: 26.8%<br>Over 65: 3.1%	Healthcare workers	26	Job:<br>Physicians: 21.06%<br>Pharmacists: 24.5%<br>Nurses: 18.1%<br>Assistant nurses: 10.65%<br>Midwives: 1.8%<br>Physiotherapist: 1.2%<br>Others (administrative staff, laboratory staff, research staff): 22.7%	25.9	N
Goldman et al 2020 (24)	Multiple countries	XS Online survey	2785	Child's mean age / SD: 7.6 /5.1<br>Guardian's mean age / SD: 39.4 / 7.86	Adolescents and caregivers	Father: 24.4<br>Child Male: 51.7	More than high school education: 76.9%	32.4^	N
Goldman et al 2020 (25)	Multiple countries	MM Online survey with qualitative component#	1541	Child's median age: 7.5 / SD 4.98CG median age: 39.9 / SD 7.58	Children and adolescents	Child gender male: 52.2<br>CG father: 25.5	Caregivers with more than high school education n=1217 (80.2%) [available total data points n=1517]	33^	N
Graffigna et al 2020 (104)	Italy	XS NS	1004	44/ 14	General public	49.1	No high school: 12.5%<br>High school: 60.0%<br>University or higher: 27.5%	41.4	Y
Grüner et al 2020 (36)	Germany	XS NS	2077	Not specified	Students and general public	Not specified	NS	HCW: 16.9	N

								HC students: 20.19<br>Non-HC students: 14.33	
Guidry et al 2020 (85)	USA	XS Online survey	788	45.9 / 17.15	General public	50	NS	40.1	Y
Head et al 2020 (68)	USA	XS Online survey	3159	46.9/ 16.8	General public	47.2	Less than high school graduate, HS graduate, GED: 23.2%<br>Some college/Associate's degree: 28.8%<br>Bachelor's degree: 29.6%<br>Graduate school: 18.3%	33.7	Y
Hoke et al 2021 (89)	USA	XS Online survey	350	Ages not specified<br>Ages as school nurse(%):<br>0–5: 24.9<br>6–15: 38.0<br>16 and more: 34.3<br>No response: 2.9	Healthcare workers	0	High school diploma or equivalent: 1.7%<br>Associate's degree: 4.9%<br>Bachelor's degree: 43.1%<br>Master's degree or higher: 50.3%	15.1	N
Hughes et al 2020 (106)	U.K.	XS Online survey	406	29 / 8.84	General public	28.6	NS	Acceptance in continuum scale max of 7 5.22 (SD 1.75)	N
Hursh et al 2020 (37)	USA	XS Online survey	534	41.9 / 13.4	General public	49	NS	NS	N
Jeffs et al 2020 (86)	New Zealand	XS Online survey	1191	39.9 ((no SD), range: 20-62)	General public	Not specified	Completed high school (12%), Trade certificate/diploma	30.4^	Y

							(13.3%), Bachelor's degree (31.4%) and Post-graduate degree (42.7%)		
Jung et al 2020 (102)	USA	MM: longitudinal and controlled experiments via online platforms	560	Median: 45	General public	48.5	Not specified	N.A.	NS
Karlsson et al 2020 (38)	Finland	XS Online survey	2355	Study 1: mean 37.9 (SD: 4.75, range: 23 to 55)<br>Study 2: mean not stated, but had more respondents who were between 50 and 65 years old than Study 1<br>Study 3: mean 47.7 (SD: 13.11, range: 18-100)	General public	Study 1: 21.2<br>Study 2: 30.7<br>Study 3: 19.1	NS	25.2 (mean of 3 hesitancies studies)	Y
Khubchandani et al 2021 (39)	USA	XS Online survey	1878	18–25 years: 19%<br>26–40 years: 44%<br>41–60 years: 28%<br>≥ 61 years: 9%	General public	48	≤ High school: 6%<br>Some college education: 17%<br>Bachelor’s degree: 48%<br>≥ Master’s degree: 29%	22	N
Klein et al 2021(18)	USA	XS Online survey	800	Not specified	General public	Not specified	NS	NS (data not provided by author)	NS
Kociolek et al 2021 (40)	USA	MM	4448	≤40: (n=2,312)<br>≥41 (n=1,855)	Healthcare workers	17.1	Job:<br>Nonclinical: 36.3%<br>Clinical: 57.7	18.9	Y

Kourlaba et al 2021 (100)	Greece	MM XS online CATI (n=502) AND CAWI (n=502)	1004	41.7 / 17.7	General public	51	Primary school: 6.8%<br>Middle school: 33.4%<br>College and above: 59.8%	42.3	Y
Kreps et al 2020 (41)	USA	MM embedded choice based conjoint analysis and XS online survey	1971	18-29 : 23%<br>30-44: 29%<br>45-59: 25%<br>60 and more: 23<br><br>Median age: 43 (IQR: 30 - 58)	General public	49	Less than high school: 2%<br>High school/GED: 21%<br>Some college: 30%<br>4-y College degree: 24%<br>Graduate school: 24%	44	Y
Kuter et al 2020 (42)	USA	XS Online survey	12034	Less than 40: 51%<br>40 - 64: 39.2%<br>65 and older: 3.6%<br>Unknown: 6.3%	Healthcare workers	21	Less than Bachelor's Degree: 12.6%<br>Bachelor's or Master's Degree: 56.5%<br>Postgrad degree: 25.8%<br>Unknown: 5.1%<br>Direct patient contact: 53.9%<br>Some patient interaction: 8.9%<br>No patient interaction: 25.3%<br>Other/Unknown: 11.9%	36.3	Y
Kwok et al 2021 (92)	Hong Kong	XS Online survey	1205	40.79 / 10.47	Healthcare workers	10	Degree holder: 32.7%	37	N
C. La Vecchia et al 2020 (69)	Italy	XS Online survey using CAWI	1055	15-34: 23.8%<br>35-54: 34.1%<br>55 and more: 42.1%	General public	48.2	NS	46	N
E. A. Largent et al 2020 (75)	USA	XS Online survey	2730	Not specified	General public	45.9	Less than bachelor degree: 1155 (42.4%)	38.6	N

							Bachelor degree or higher: 1569 (57.6%)		
C. A. Latkin et al 2021 (43)	USA	XS Combined online and Telephone survey	1056	18 to 29 years old: 71 (6.8%)<br>30 to 39 years old: 147 (14.1%)<br>40 to 59 years old: 373 (35.8%)<br>60 to 64 years old: 118 (11.3%)<br>65 years old or older: 334 (32.0%)	General public	29.9	High school and below: 203 (19.5%)<br>Some college and above: 840 (80.5%)	46.4	Y
Loomba et al 2021 (109)	Multiple countries	RCT via online platform	8001	18-44 years: 45.5% (intervention), 63.2% (control).<br>45 and above: 54.5% (intervention), 36.8% (control).	General public	42.3 (intervention)<br>45.7 (control)	Level 0-2: 51.9% (intv), 49.6% (cntl).<br>Level 3 and 4: 41.3% (intv), 43% (cntl).	UK 45.9<br>U.S. 57.5	Y
V. C. Lucia et al 2020 (95)	USA	XS Online survey	168	Not specified	University students	43	All undergraduates	24.6	N
A. A. Malik et al 2020 (44)	USA	XS Online survey	672	18-24 years: 73 (11%)<br>25-34 years: 107 (16%)<br>35-44 years: 141 (21%)<br>45-54 years: 95 (14%)<br>55+ years: 256 (38%)	General public	42	No High School: 10 (2%)<br>High School: 162 (24%)	33	Y
M. L. Manning et al 2021 (45)	USA	XS Online survey	1212	<30 years: 710 (69.1%)<br>31-40 years: 220 (21.4%)<br>41-50 years: 68 (6.6%)<br>51-61 years: 22 (2.1%)<br>60 and greater: 2 (0.2%)<br>Prefer not to disclose: 5 (0.5%)	University staff and students	Students: 11.3<br>FT Faculty: 5.1<br>Adjuncts: 12.4	Students<br>Undergraduate: 486 (47.3%)<br>Graduate: 423 (41.2%)<br>DNP: 108 (10.5%)<br>Others: 10 (1.0%)<br><br>Faculty<br>Undergraduate: 41 (52.6%)<br>Graduate: 26 (33.3%)<br>DNP: 6 (7.7%)	53.7	Y

				FT Faculty<br><30 years: 0 (0%)<br>31-40 years: 15 (20.8%)<br>41-50 years: 15 (20.8)<br>51-61 years: 25 (34.7%)<br>60 and greater: 11 (15.3%)<br>Prefer not to disclose: 6 (8.3%)<br><br>Adjuncts<br><30 years: 4 (3.8%)<br>31-40 years: 45 (42.9%)<br>41-50 years: 22 (21.0%)<br>51-61 years: 17 (16.2%)<br>60 and greater: 14 (13.3%)<br>Prefer not to disclose: 3 (2.9%)			Others: 5 (6.4%)<br><br>Adjuncts<br>Undergraduate: 74 (70.5%)<br>Graduate: 27 (25.7%)<br>DNP: 2 (1.9%)<br>Others: 2 (1.9%)		
McPhedran et al 2021 (70)	United Kingdom	XS Online survey	1501	16-34 years: 466 (31%)<br>35-54 years: 480 (32%)<br>55+ years: 555 (37%)	General public	49	NS	NS - discrete choice experim ent	NS
A. R. Mercadante et al 2021 (71)	USA	XS Online survey	525	18-29 years: 110 (21.0%)<br>30-49 years: 172 (32.8%)<br>50-69 years: 168 (32.0%)<br>70 or older: 74 (14.1%)	General public	50.1	Some high school: 24 (4.6%)<br>High school diploma: 143 (27.2%)<br>Some college: 143 (27.2%)<br>Associate degree: 50 (9.5%)<br>Bachelor degree: 105	33.3	N

				Not disclosed: 1 (0.2%)			(20.0%)<br>Master degree: 56 (10.7%)<br>I do not want to disclose: 4 (0.8%)		
F. Momplaisir et al 2021 (27)	USA	MM with focus group discussion and questionnaire via zoom#	24	46.3 (20, 63)	General public	26.3	NS	NS	NS
M. Motta et al 2021 (111)	USA	MM with Online survey	5940	Not specified	General public	Not specified	NS	NS – discrete choice experiment.	NS
Muqattash et al 2020 (87)	UAE	XS Online survey	1109	[18 to 25]: 12.9%<br>[26 to 35]: 28.0%<br>[36 to 45]: 39.4%<br>[45 and over]: 19.8%	General public	27.9	None: 3.9%<br>High School: 10.2%<br>Diploma: 11.3&<br>Graduate: 59.1%<br>Postgraduate: 15.6%	52.8	N
J. Murphy et al 2021 (84)	Multiple countries	XS Online survey	4077	Ireland<br>18-24: 11.1%<br>25-34: 19.2%<br>35-44: 20.6%<br>45-54: 15.9%<br>55-64: 21.0%<br>65+: 12.2%<br><br>UK<br>18-24: 12.1%<br>25-34: 18.8%<br>35-44: 17.4%<br>45-54: 20.2%<br>55-64: 17.2%<br>65+: 14.2%	General public	Ireland: 48.2<br>UK: 48.3	Ireland<br>No qualification: 1.2%<br>Finished Mandatory schooling: 6.4%<br>Finished secondary school: 22.4%<br>Undergraduate degree: 22.5%<br>Postgraduate degree: 19.8%<br>Other technical qualification: 27.9%<br><br>UK<br>No qualifications: 2.9%<br>O-level / GCSE or similar: 19.0%	32.4	Y

							A level or similar: 18.1%<br>Diploma: 5.6%<br>Undergraduate degree: 28.2%<br>Postgraduate degree: 15.6%<br>Technical qualification: 9.3%<br>Other: 1.3%		
Neumann-Böhme et al 2020 (107)	Multiple countries	XS Online survey	7664	Not specified	General public	Not specified	NS	26.1	Y
K. H. Nguyen et al 2021 (46)	USA	XS Online survey	3541	18-49 years old: 39.5%<br>50-64 years old: 42.0%<br>>=65 years old: 29.8%	General public	33.8	High school and below: 47.0%<br>Some college or college graduate: 35.8%<br>Above college graduate: 23.8%	Mean of 2: 35.1	N
Niankara et al 2020 (82)	UAE	XS Online survey	1109	18 to 25 years old: 143 (13%)<br>26 to 35 years old: 310 (28%)<br>36 to 45 years old: 437 (39%)<br>45 years old and above: 219 (20%)	General public	27.86	High school and below: 156 (14%),<br>Diploma: 125 (11%),<br>Graduate: 655 (59%),<br>Postgraduate: 173 (16%)	77.9	N
A. A. Olagoke et al 2021 (76)	USA	XS Online survey	501	NS	General public	44.71	Less than high school/High school: 70 (14.03%)<br>Some college: 161 (32.26%)<br>College or more: 268 (53.71%)	Acceptance continuum scale maxx score of 5: 4.24 (1.04)	N
L. Palamenghi et al 2020 (99)	Italy	XS Online survey	1972	NS	General public	NS	NS	41	Y
K. Pogue et al 2020 (22)	USA	XS Online survey	316	Less than 18: 7 (2.16%)	General public	49.07	NS	31.5	Y

				18-25: 40 (12.4%)<br>26-35: 59 (18.2%)<br>36-45: 102 (31.5%)<br>46-55: 11 (3.4%)<br>>55: 105 (32.4%)				Mean 21.5^	
G. Prati et al 2020 (96)	Italy	XS Online survey	624	Not specified	General public	NS	NS	15.2	Y
R. Priori et al 2021 (47)	Italy	Case control online survey	1175	Not specified	Patients with autoimmune conditions	NS	NS	Rheumato: 45.1%<br>Healthy controls: 17.7	Y
P. L. Reiter et al 2020 (48)	USA	XS Online survey	2006	18-29 years old: 313 (16%)<br>30-49 years old: 657 (33%)<br>50-64 years old: 532 (27%)<br>65 years and older: 504 (25%)	General public	43	Less than high school degree: 105 (5%)<br>High school degree: 589 (29%)<br>Some college: 629 (31%)<br>College degree or more: 683 (34%)	31	Y
Romer et al 2020 (72)	USA	Longitudinal prospective study Online survey	1890	(18–29): (20.5 - 20.7)%<br>(30–44): (23.9 - 24.1)%<br>(45–59): (24.9 - 25.2)%<br>60+: (30.0 - 30.7)%	General public	Wave 1: 45.6<br>Wave 2: 44.3	(Wave 1 - wave 2)%<br>High school: (32.6 - 32.6)%<br>Some College: (47.8 - 48.3)%<br>Post-Grad: (19.6 - 30.1)%	Mean of 2: 20.2	N
J. B. Ruiz et al 2021 (49)	USA	XS Online survey	804	18-24: 118 (14.7)<br>25-34: 146 (18.2)<br>35-44: 150 (18.7)<br>45-54: 135 (16.8)<br>55-64: 114 (14.2)<br>>=65: 141 (17.5)	General public	46.4	HS or less: 160 (19.9%)<br>Some college / associates degree: 221 (27.5%)<br>4 years college degree: 236 (29.4%)<br>Post-graduate degree: 187 (23.3%)	37.8	Y
Schmelz et al 2020 (50)	Germany	XS Online survey	4799	48 / 16	General public	49	NS	22	Y

Schwarzinger et al 2021 (51)	France	MM Online survey with embedded discrete choice experiment	1942	18-24: 257 (13.2%)<br>25-34: 391 (20.1%)<br>35-44: 427 (22%)<br>45-54: 448 (23.1%)<br>55-64: 419 (21.6%)	General public	48.9	Some high school: 854 (44%) High school graduate: 420 (21.6%) University graduate: 668 (34.4%)	28.8	N
H. Seale et al 2021 (73)	Australia	XS Online survey	1143	18-29 years old: 295 (20.8%)<br>30-49 years old: 508 (35.8%)<br>50-69 years old: 419 (29.5%)<br>70+ years old: 198 (13.9%)	General public	47.7	Year 10 or below: 161 (11.3%)<br>High school: 235 (16.5%)<br>Trade/apprenticeship/cert: 483 (34%)<br>University: 541 (38.1%)	20	Y
Shaw et al 2021 (52)	USA	MM Online survey and 2 opended questions	5308	42.5	Healthcare workers	26	58.5% provide direct patient care; among these 32.2% provided care for patients with COVID-19	42.3	Y
R. Shekhar et al 2021 (53)	USA	XS Online survey	3479	18-30 years old: 816 (23%)<br>31-40 years old: 1061 (30%)<br>41-50 years old: 686 (20%)<br>51-60 years old: 571 (16%)<br>61-70 years old: 326 (9.4%)<br>>70 years old: 19 (0.5%)	Healthcare workers	25	No formal education: 1 (<0.1%)<br>High school graduate, diploma or the equivalent (for example GED): 46 (1.3%)<br>Some college credit, no degree: 169 (4.9%)<br>Trade / technical / vocational training: 111 (3.2%)<br>Associate degree: 364 (10%)<br>Bachelor degree: 1046 (30%)<br>Master degree: 606 (17%)<br>Professional degree: 297 (8.5%)	64	Y

S. M. Sherman et al 2020 (54)	United Kingdom	XS Online survey	1500	Not specified	General public	48.6	Doctorate degree: 839 (24%) Degree equivalent or higher: 789 (52.6%) Degree equivalent or higher: 604 (46.9%) Prefer not to say: 7 (0.5%)	36	N
P. Sprengholz et al 2021 (93)	Italy	XS Online survey	1349	47.31 years old (SD: 14.02)	General public	49.4	NS	Acceptamce Continuum scale max score of 7: 4.43 (SD=2.33).	N
Szilagyi et al 2021 (55)	USA	XS Online survey	8167	address-based sampling to allow statistical inferences	General public	NS but mentioned address-based sampling to allow statistical inferences	NS but mentioned address-based sampling to allow statistical inferences	Mean of 2: 34.9	Y
B. Szmyd et al 2021 (108)	Poland	XS Online survey	1971	Median age (Medical students): 21 (20-24) Median age (non-medical students): 20 (19-22)	University students	Medical students: 35.23 Non-medical students: 56.70	Undergraduates: 100%	MS: 8.01 Non-MS: 40.58	N
Taylor et al 2020 (90)	Multiple countries	XS Online survey	3674	mean age was 53 years (SD = 15 years)	General public	57	Completed full or partial college: 82%	Americans:25 Canadians: 20	N
Underschultz et al 2021 (88)	Canada	XS Online survey	1593	16 - 29: 52% 30 - 49: 27% 50+: 21%	General public	29	High School and below: 13% Bachelor's degree (in	7	N

							process of or completed): 56%<br>Post-graduate degree (in process of or completed): 31%		
K. T. Unroe et al 2021 (56)	USA	XS Phone and email survey	8243	16-24 years old: 1044 (12.7%)<br>25-40 years old: 3053 (37.0%)<br>41-60 years old: 3204 (38.9%)<br>>60 years old: 938 (11.4%)	Healthcare workers	12.6	NS	55.1	Y
P. Verger et al 2021 (57)	Multiple countries	XS Online survey	2678	18-39: 920 (34.4%)<br>40-59: 1250 (46.7%)<br>>=60: 508 (19.0%)	Healthcare workers	30.75	NS	28.4	Y
K. Wang et al 2020 (58)	Hong Kong	XS Online survey	806	18-29 years old: 174 (21.6%)<br>30-39 years old: 251 (31.1%)<br>40-49 years old: 218 (27.1%)<br>50+ years old: 163 (20.2%)	Healthcare workers	12.50	NS	60	Y
K. Wang et al 2021 (59)	Hong Kong	XS Online survey	1196	18-29 years old: 161 (16.1%)<br>30-39 years old: 225 (22.5%)<br>40-49 years old: 247 (24.7%)<br>50-59 years old: 243 (24.3%)<br>60+ years old: 124 (12.4%)	General public	53.90	Below high school: 45 (4.5%)<br>High School: 255 (25.5%)<br>Preparatory: 246 (24.6%)<br>University or above: 454 (45.4%)	Mean of 2: 41.2	N
J. K. Ward et al 2020 (60)	France	XS Online survey	5018	<35 years old: 33.1%<br>35-64 years old: 25.8%<br>>64 years oldL 10.7%	General public	20.60	Lower than high school degree: 23.6%<br>High school degree: 27.5%<br>Two or three year	24	N

							undergraduate degree: 23.8%<br>Higher than Bachelor degree: 21.4%		
W. Wardha et al 2021*	USA	XS On site face to face administered questionnaire	220	Not specified	General public	NS	NS	35	Y
Williams et al 2020 (97)	U.K.	MM with XS Online survey and opened ended questions	527	59.5 / 16	General public	43	High School: 17.3%<br>College: 31.2%<br>University: 29.1%<br>Postgraduate: 22.3%	14.4	Y
Williams et al 2021 (83)	Scotland	XS Online survey	3436	Time 1:<br>18-49 years old: 1847 (53.8%)<br>50+ years old: 1578 (45.9%)<br><br>Time 2:<br>18-49 years old: 974 (48.3%)<br>50+ years old: 1034 (51.5%)	General public	TIme 1: 19.4<br>Time 2: 17.9	Time 1<br>No quals / left school 16:<br>168 (4.9%)<br>High school / college: 780 (22.7%)<br>University: 2435 (70.9%)<br><br>Time 2<br>No quals / left school 16:<br>77 (3.8%)<br>High school / college: 439 (21.8%)<br>University: 1467 (72.8%)	24	Y
Wong et al 2021 (91)	Hong Kong	XS Telephone interview	1200	18-24 years old: 71 (5.9%)<br>25-34 years old: 68 (5.7%)<br>35-44 years old: 112 (9.3%)	General public	28.7	Primary or below: 442 (36.8%)<br>Secondary: 474 (39.5%)<br>Tertiary or above: 275 (22.9%)	57.8	Y

				45-54 years old: 124 (10.3%)<br>55-64 years old: 261 (21.8%)<br>>=65 years old: 562 (46.8%)<br>Refused to answer: 2 (0.2%)			Refused to answer: 9 (0.8%)		
T. Yoda et al 2021 (61)	Japan	XS Online survey	1100	44.8	General public	53.1	NS	34.3	Y
Hamel et al 2020 (77)	USA	MM: Combined stratified, dual-frame (landline and cell phone) random digit dial (RDD) telephone sample. Computer-assisted telephone interviews were conducted with the RDD sample.	1769	Not specified	General public	49	NS	34	N
Survey Healthcare Globus 2020**	Multiple countries	XS Online survey	484	Not specified	Healthcare workers	NS	NS	NS	NS

Abbreviations: NS – not specified; XS – cross sectional; MM – mixed methods; CAWI – Computer Assisted Web Interview; CATI – Computer Assisted Telephone Interview; SD – standard deviation; IQR: Interquartile range

# – Qualitative studies with well described methodologies

* – not full text

** – explored healthcare workers on their opinions of what could be the reasons of vaccine hesitancy in their patients

^ – refers to guardians’ hesitancy towards their wards
